# Sleeping Late Increases the Risk of Myocardial Infarction in the Middle-Aged and Older Populations

**DOI:** 10.3389/fcvm.2021.709468

**Published:** 2021-09-24

**Authors:** Yajuan Fan, Yanhua Wu, Yuan Peng, Binbin Zhao, Jian Yang, Ling Bai, Xiancang Ma, Bin Yan

**Affiliations:** ^1^Department of Psychiatry, the First Affiliated Hospital of Xi'an Jiaotong University, Xi'an, China; ^2^Department of Clinical Research Centre, the First Affiliated Hospital of Xi'an Jiaotong University, Xi'an, China; ^3^Department of Cardiology, the First Affiliated Hospital of Xi'an Jiaotong University, Xi'an, China

**Keywords:** bedtime, wake-up time, sleep midpoint, myocardial infarction, sleep heart health study

## Abstract

**Objective:** Sleep has a significant influence on the incidence of myocardial infarction (MI). The purpose of this study was to investigate the association between sleep timing including bedtime, wake-up time and sleep midpoint, and the incidence of MI.

**Methods:** A total of 4,576 patients (2,065 men, 2,511 women; age 63.4 ± 11.0 years) were selected from the Sleep Heart Health Study. Sleep timings on weekdays and weekends were recorded or calculated based on the sleep habits questionnaire completed by the participants at baseline. Bedtime was divided into 10:00 PM and before, 10:01 PM−11:00 PM, 11:01 PM−12:00 AM, and later than 12:00 AM. Cox proportional hazards regression analysis was used to examine the relationship between sleep timings and MI.

**Results:** Participants with a weekday bedtime later than 12:00 AM, between 11:01 PM−12:00 AM, and 10:00 PM or before had a higher incidence of MI than those with a bedtime between 10:01 PM and 11:00 PM (9.2% vs. 7.0% vs. 6.9% vs. 5.1%, respectively; *P* = 0.008). Multivariable Cox regression analysis showed that sleeping on weekdays later than 12:00 AM was associated with an increased risk of incident MI after adjusting for potential covariates (hazard ratio, 1.628; 95% confidence interval, 1.092–2.427; *P* = 0.017). However, there was no significant association between late bedtime on weekends and MI. In addition, no significant association of late wake-up time and delayed sleep midpoint on both weekdays and weekends with the incidence of MI was observed.

**Conclusion:** Sleeping late on weekday (>12:00 AM) independently increased the risk of MI. This finding emphasizes the importance of a proper bedtime for the maintenance of the health of the cardiovascular system.

## Introduction

Myocardial infarction (MI) is the most serious manifestation of coronary artery disease, and affects more than 7 million people worldwide each year. It is usually defined as myocardial cell death caused by substantial continuous ischemia due to an imbalance between the oxygen supply and demand (1, 2). In recent decades, the increased use of pharmacotherapy and evidence-based therapies has significantly reduced the mortality rate of coronary heart disease, but the global burden is still enormous for individuals and societies (3, 4).

Sleep is a necessary physiological activity that plays an important role in maintaining health as well as preventing cardiometabolic disease (5). Insufficient sleep can lead to reduced carbohydrate tolerance and increased sympathetic tone, which increase the risk of insulin resistance, obesity, hypertension and may further contribute to MI (6, 7). Previous studies showed that self-reported sleep duration <6 h was a potential pathogenic factor for MI (8). Genetically determined short sleep duration (<6 h) was also a potential causal risk factor of MI (9). Self-reported sleep duration could be calculated as the length of time from bedtime to wake-up time. Several studies have shown that the sleep timing such as bedtime and wake-up time were correlated with MI risk factors including diabetes mellitus, obesity, and physical activity (10–12).

However, the association between sleep timing (including bedtime, wake-up time and sleep midpoint) and MI remains unknown. Therefore, we conducted this study to investigate the association between sleep timing and MI on the basis of a decade-long follow-up dataset from the Sleep Heart Health Study (SHHS).

## Methods

### Participants

**Figure 1:**
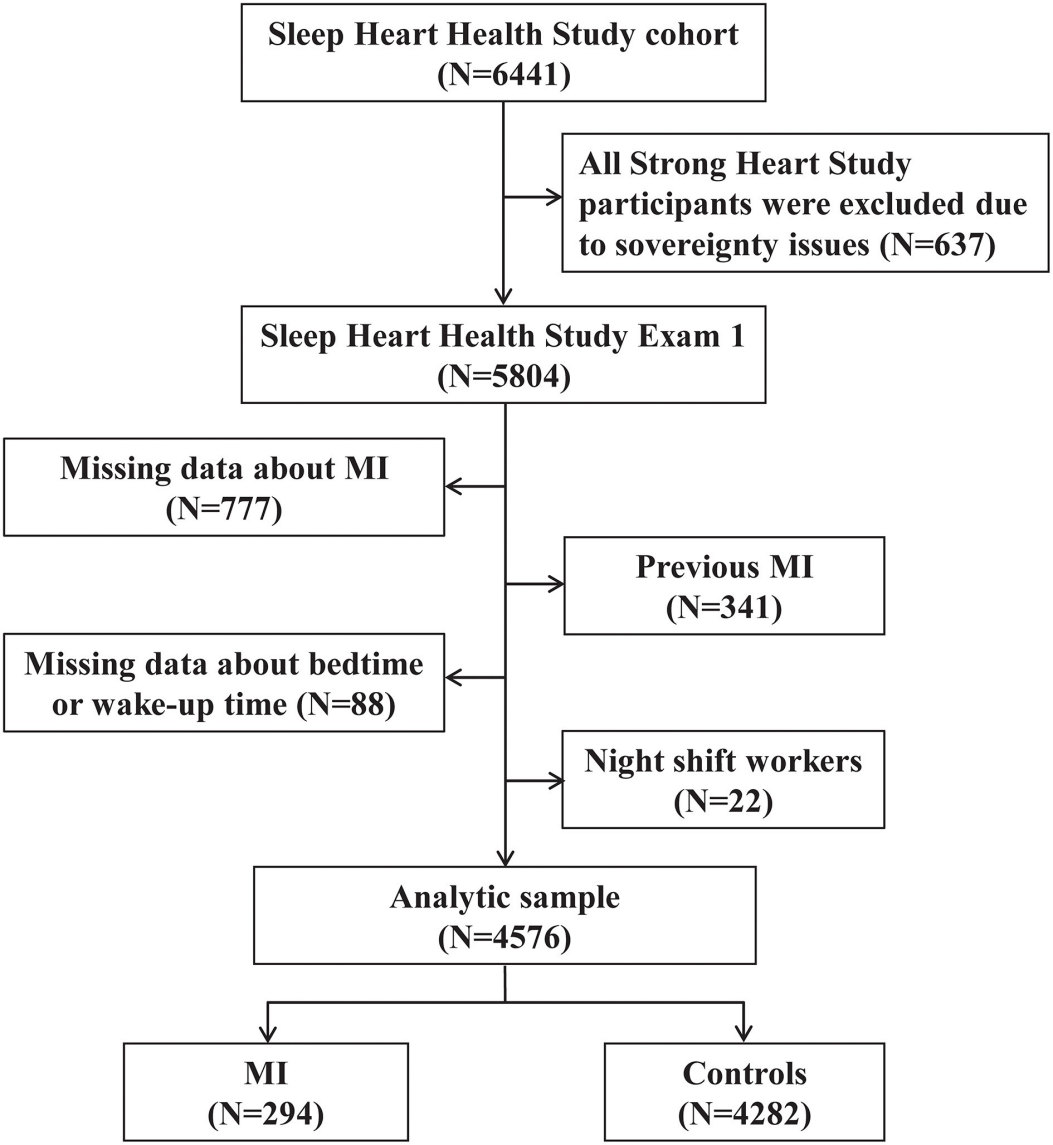
Flow diagram of participant selection.

All participants in our study were selected from the SHHS (ClinicalTrials.gov identifier: NCT00005275). The SHHS is a community-based, multi-center, prospective cohort study that was recruited from nine existing epidemiological studies (13). All participants enrolled in the SHHS were aged 40 years or older, and filled out sleep habit questionnaire and underwent polysomnography monitoring between 1995 and 1998. Cardiovascular diseases such as MI was observed from baseline until followed up to 2011. The protocol was approved by the institutional review board of each participating institution (Boston University, Case Western Reserve University, Johns Hopkins University, Missouri Breaks Research, Inc., New York University Medical Center, University of Arizona, University of California at Davis, University of Minnesota, Clinical and Translational Science Institute, University of Washington) (14). The data quality assurance and control system was used in each parent study when collected data. All participants provided written consent for the follow-up and health examinations. We obtained the SHHS data after signing an agreement with the Brigham and Women's Hospital in Boston, Massachusetts, USA. The participants were excluded if (1) there were missing data about MI (*n* = 777); (2) they previously experienced MI (*n* = 341); (3) missing data about the bedtime and wake-up time (*n* = 88); and (4) they had day-night reversal (*n* = 22). Finally, 4,576 participants were included in the study (Figure 1).

### Data Collection

Information about the sleep timing including bedtime and wake-up time was acquired by asking the participants to complete the Sleep Habits Questionnaire at the baseline. Bedtimes were recorded using questions, such as “At what time do you usually fall asleep on weekdays and weekends, respectively (hour, minute, AM or PM)?” A similar question was also used to record the wake-up times on weekdays and weekends. In our pre-analysis, we found a U-sharp relationship between weekday bedtime and MI based on the restricted cubic spline analysis. Individuals with weekday bedtime at 10:01 PM−11:00 PM had the lowest incidence MI when compared with 12:00 AM and later, 11:01 PM−12:00 AM and 10:00 PM and before (Supplementary Figure 1). Combining the pre-analysis and previous studies, the bedtime was classified into 10:00 PM and before, 10:01 PM−11:00 PM, 11:01 PM−12:00 AM, and 12:00 AM and later in this study (15, 16). Wake-up time was categorized into 8:00 AM and later, 7:01 AM−8:00 AM, 6:01 AM−7:00 AM, and 6:00 AM and earlier. Sleep midpoint refers to the intermediate time point between bedtime and wake-up time. Self-reported sleep duration was calculated as the length of time from bedtime to wake-up time. The apnea-hypopnea index (AHI) was calculated from all apnea and hypopnea events that were accompanied by at least a 4% drop in the oxygen saturation per hour of sleep based on the baseline polysomnography records.

The diagnosis of MI relied on a combination of chest pain, electrocardiogram (ECG) tracings and myocardial enzyme profiles at all sites. The MI recorded in this study was referred to as the first occurrence between the SHHS baseline (1995 to 1998) and end of follow-up time (2011). Participants' data including age, sex, race, body mass index (BMI), smoking status, alcohol use, history of hypertension, diabetes mellitus, and MI were obtained from the SHHS baseline examination.

### Statistical Analysis

Continuous variables are presented as means ± standard deviations (SD) and categorical variables as numbers (percentages) which were compared using the Student's *t*-test and chi-square test, respectively. The follow-up time was defined as the time interval from the baseline until the first diagnosis of MI or the time interval from the baseline until the last review of the clinical records of the participants without the disease. Survival curves were generated using the Kaplan-Meier product-limit method and compared using the Mantel log-rank test (17). Multivariable Cox proportional hazards regression model was used to examine the relationship between sleep timing (bedtime, wake-up time, sleep midpoint) and MI incidence after adjusting for age, sex, race, BMI, smoking status, alcohol use, hypertension, diabetes mellitus, AHI and self-reported sleep duration. Furthermore, interaction analysis was performed by comparing the models with and without multiplicative interaction terms (sleep timing × sleep duration). All statistical analyses were performed using the SPSS software package (version 24.0; SPSS Corporation, Chicago, USA) and R version 3.6.0. All statistical tests were two-sided, and a *P*-value < 0.05 was considered statistically significant.

## Results

### Demographic and Clinical Characteristics

The study included 2,065 men and 2,511 women, and most of the study population was white (87.0%). A total of 294 (6.4%) individuals with MI were observed during the 10.6 ± 3.2 years' follow-up. Participants with MI were older and more likely to be men. Moreover, the MI group had a higher prevalence of smoking, diabetes mellitus, and hypertension than did the non-MI group (Table 1).

**Table 1:** Sleep characteristics in participants with or without MI.

**Variables**	**Total (*n* = 4,576)**	**Incident MI (*n* = 294)**	**No MI (*n* = 4,282)**	***P*-value**
Age, year	63.4 ± 11.0	71.1 ± 10.0	62.9 ± 10.9	<0.001
Sex, *n* (%)				<0.001
Men	2,065 (45.1)	176 (59.9)	1,889 (44.1)	—
Women	2,511 (54.9)	118 (40.1)	2,393 (55.9)	—
Race, *n* (%)				0.004
White	3,979 (87.0)	245 (83.3)	3,734 (87.2)	—
Black	303 (6.6)	33 (11.2)	270 (6.3)	—
Other	294 (6.4)	16 (5.5)	278 (6.5)	—
Body mass index, kg/m^2^	28.3 ± 5.1	28.3 ± 4.6	28.3 ± 5.1	0.968
Smoking status, *n* (%)				0.001
Current smoker	437 (9.6)	30 (10.2)	407 (9.5)	—
Former smoker	1,982 (43.4)	156 (53.3)	1,826 (42.8)	—
Never smoker	2,143 (47.0)	107 (36.5)	2,036 (47.7)	—
Alcohol use, *n* (%)				0.019
At least 1 drink per day	1,871 (43.7)	106 (37.1)	1,765 (44.2)	—
None	2,411 (56.3)	180 (62.9)	2,231 (55.8)	—
Diabetes mellitus, n (%)	308 (6.7)	44 (15.0)	264 (6.2)	<0.001
Hypertension, *n* (%)	1,730 (37.8)	169 (57.5)	1,561 (36.5)	<0.001
Sleep duration, *n* (%)				0.055
<6 h	322 (7.0)	26 (8.8)	296 (6.9)	—
6–8 h	3,341 (73.0)	197 (67.0)	3,144 (73.4)	—
>8 h	913 (20.0)	71 (24.2)	842 (19.7)	—
AHI, *n* (%)				0.018
<5.0 events/h	2,272 (49.7)	121 (41.2)	2,151 (50.2)	—
5–14.9 events/h	1,379 (30.1)	98 (33.3)	1,281 (29.9)	—
15-29.9 events/h	592 (12.9)	49 (16.7)	543 (12.7)	—
≥30 events/h	333 (7.3)	26 (8.8)	307 (7.2)	—
Follow-up time, year	10.6 ± 3.2	6.0 ± 3.3	10.9 ± 2.9	<0.001

*AHI, apnea hypopnea index; Results are presented as mean ± standard deviation or n (%). The P-values represent the difference between two groups*.

### Association of Late Bedtime With Incident MI

**Figure 2:**
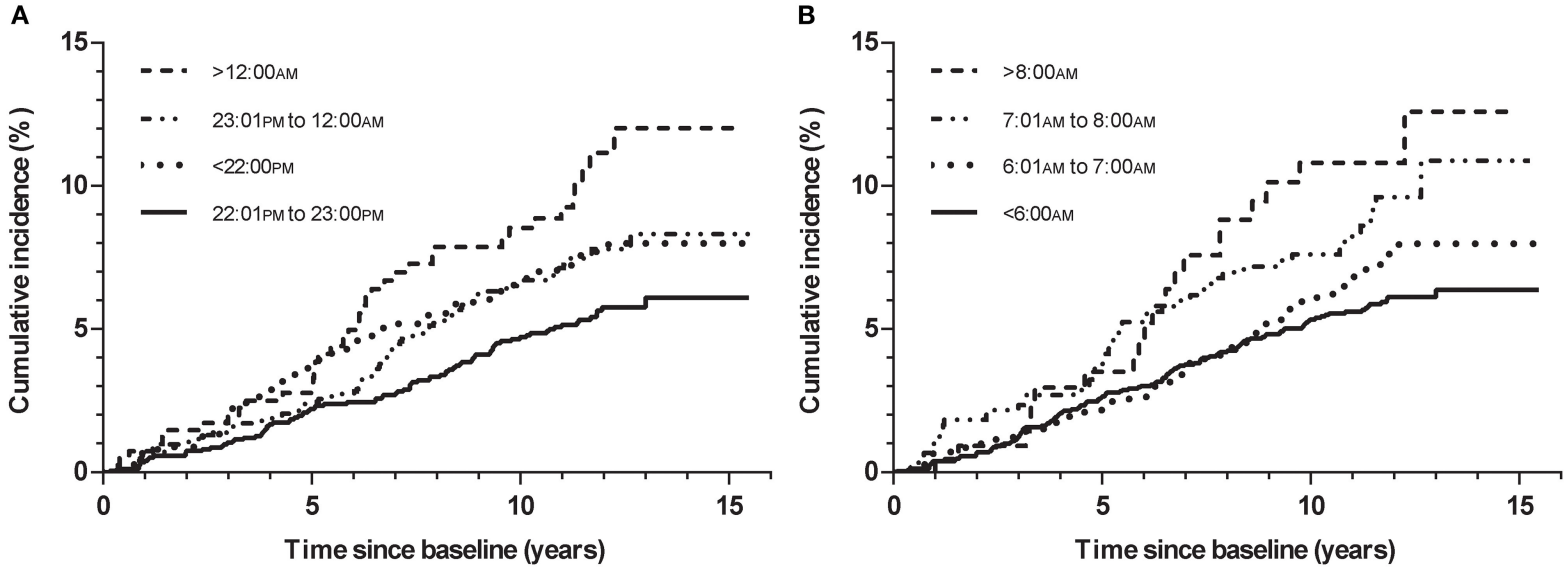
Kaplan-Meier plots of cumulative risk for MI stratified by different categories of bedtime and wake-up time on weekday. **(A)**: Weekday bedtime and MI; **(B)**: Weekday wake-up time and MI.

Individuals with a weekday bedtime >12:00 AM (9.2%) had the highest incidence of MI and morbidity rate than those with a bedtime at 11:01 PM−12:00 AM (7.0%), 10:01 PM−11:00 PM (5.1%), and ≤10:00 PM (6.9%) (Table 2). The Kaplan-Meier survival curves also supported these findings (Figure 2A). Univariate Cox regression analysis revealed that individuals with a weekday bedtime at >12:00 AM had a higher risk of MI than those with a bedtime at 10:01 PM−11:00 PM [hazard ratio (HR) 2.011, 95% confidence interval (CI) 1.376–2.939; *P* < 0.001]. After adjusting for age, sex, race, BMI, smoking status, alcohol use, hypertension, diabetes mellitus, AHI, and sleep duration, weekday bedtime at >12:00 AM was independently associated with an increased risk of incident MI (HR 1.628, 95% CI 1.092–2.427; *P* = 0.017) (Table 2).

**Table 2:** Hazard ratios and 95% CIs for bedtime associated with MI on weekday.

	**Total**	**>12:00_**AM**_**	**11:01_**PM**_ to 12:00_**AM**_**	**10:01_**PM**_ to 11:00_**PM**_**	**≤10:00_**PM**_**
No. of subjects, *n*	4,576	413	1,243	1,777	1,143
Person-years	4,8454.8	4,052.7	13,196.7	19,272.5	11,932.9
Events, *n* (%)	294 (6.4)	38 (9.2)	87 (7.0)	90 (5.1)	79 (6.9)
Morbidity rate^§^	6.1	9.4	6.6	4.7	6.6
**Hazard ratio for weekday bedtime**
Univariate models		2.011 (1.376–2.939)^&^	1.407 (1.048–1.890)^*^	1(Ref)	1.422 (1.051–1.923)^*^
Age and sex adjusted		1.812 (1.239–2.650)^#^	1.268 (0.944–1.704)	1(Ref)	1.419 (1.049–1.921)^*^
Multivariable adjusted^a^		1.625 (1.106–2.388)^*^	1.181 (0.878–1.590)	1(Ref)	1.320 (0.974–1.789)
Multivariable adjusted^b^		1.628 (1.092–2.427)^*^	1.194 (0.885–1.611)	1(Ref)	1.277 (0.935–1.744)

*MI, myocardial infarction; 95% CI, 95% confidence interval*.

§ *Crude event rate per 1,000-person years*.

* *P < 0.05*;

# *P < 0.01*;

& *P < 0.001*.

a *adjusted for age, sex, race, BMI, smoking status, alcohol use, hypertension, diabetes mellitus, AHI*.

b *adjusted by a+ self-reported sleep duration*.

The relationship between late bedtime on weekends and MI was also explored. Although participants with late bedtime tended to have a high incidence of MI, no significant association was found in the multivariable Cox regression analysis (Supplementary Table 1).

### Association of Late Wake-Up Time and Delaying Sleep Midpoint With Incident MI

We also analyzed the effect of late wake-up time and delayed sleep midpoint on the incidence of MI. People with wake-up time on weekdays at >8:00 AM, 7:01 AM−8:00 AM, 6:01 AM−7:00 AM, and ≤6:00 AM were prone to have a gradually increasing incidence of MI (9.0% vs. 8.3% vs. 6.7% vs. 5.5%, respectively; *P* = 0.024) (Table 3 and Figure 2B). However, there was no significant association between wake-up time >8:00 AM on weekdays and MI incidence in multivariable Cox regression analysis (HR 1.553, 95% CI 0.934–2.582; *P* = 0.089). Additionally, the relationship between the sleep midpoint and MI incidence was investigated, but no significant results were found (Table 4 and Supplementary Table 1).

**Table 3:** Hazard ratios and 95% CIs for wake-up time associated with MI on weekday.

	**Total**	**>8:00_**AM**_**	**7:01_**AM**_ to 8:00_**AM**_**	**6:01_**AM**_ to 7:00_**AM**_**	**≤6:00_**AM**_**
No. of subjects, *n*	4,576	221	602	1,415	2,338
Person-years	48,454.8	2,022.1	6,042.9	15,058.1	25,331.7
Events, n (%)	294 (6.4)	20 (9.0)	50 (8.3)	95 (6.7)	129 (5.5)
Morbidity rate^§^	6.1	9.9	8.3	6.3	5.1
**Hazard ratio for weekday bedtime**
Univariate models		1.953 (1.219–3.128) ^#^	1.618 (1.167–2.242) ^#^	1.235 (0.947–1.610)	1(Ref)
Age and sex adjusted		1.657 (1.031–2.662) ^*^	1.356 (0.974–1.887)	1.112 (0.852–1.453)	1(Ref)
Multivariable adjusted^a^		1.520 (0.944–2.446)	1.384 (0.994–1.928)	1.133 (0.867–1.480)	1(Ref)
Multivariable adjusted^b^		1.553 (0.934–2.582)	1.418 (0.991–2.028)	1.162 (0.883–1.530)	1(Ref)

*MI, myocardial infarction; 95% CI, 95% confidence interval*.

§ *Crude event rate per 1,000-person years*.

* *P < 0.05*;

# *P < 0.01*.

a *adjusted for age, sex, race, BMI, smoking status, alcohol use, hypertension, diabetes mellitus, AHI*.

b *adjusted by a+ self-reported sleep duration*.

**Table 4:** Hazard ratios and 95% CIs for sleep midpoint associated with MI on weekday.

	**HR (95% CI)**	***P*-value**
Unadjusted	1.117 (0.994–1.254)	0.063
Age and gender adjusted	1.051 (0.932–1.184)	0.418
Multivariable adjusted^a^	1.037 (0.924–1.165)	0.534
Multivariable adjusted^b^	1.037 (0.924–1.164)	0.538

*MI, myocardial infarction; 95% CI, 95% confidence interval; HR, hazard ratio*.

a *adjusted for age, sex, race, BMI, smoking status, alcohol use, hypertension, diabetes mellitus, AHI*.

b *adjusted by a+ self-reported sleep duration*.

### Interaction Analysis

The interaction effect was examined by adding the multiplicative interaction terms (sleep timing × sleep duration) in the final multivariable Cox regression model to explore the association between sleep timing (including bedtime, wake-up time, and sleep midpoint) and MI. No significant interactions were observed in these analyses (Supplementary Table 2).

## Discussion

In this community-based study, we investigated the impact of sleep timing on the incidence of MI using longitudinal data with an average follow-up of 11 years. Our study established an association between the weekday bedtime and incident MI, and showed that sleeping later than 12:00 AM on weekdays independently increased the risk of MI after adjusting for potential covariates. However, late wake-up time and delayed sleep midpoint were not associated with the incidence of MI.

Sleep has been identified as a public behavioral lifestyle health which emphasizing the impact of poor sleep on the prevalence of disease (18). Sleep quality, sleep habits, sleep duration and sleep architecture were found to be closely related to cardiovascular disease (19–21). Moreover, sleeping late was found to be associated with several MI risk factors. Previous studies revealed that individuals with a delayed bed time had a low level of physical activity (11, 22). In addition, sleeping late was significantly associated with obesity among children, adolescents and adults (12, 23, 24). Our previous study also demonstrated an association between sleeping late and the increased prevalence of diabetes mellitus (10). However, no evidence regarding the impact of late bedtime on the incidence of MI has been reported. A recent study found that there was a U-shaped association between bedtime and the composite cardiovascular disease (25). We also observed the similar condition when explore the association of weekday bedtime and incident MI. In our study, individuals with weekday bedtime >11:00 PM and ≤10:00 PM were prone to have a high incidence of MI. After multivariate Cox regression analysis, our results showed that participants with weekday bedtime later than 12:00 AM had a 62.8% increased risk of MI than those with a bedtime between 10:01 PM−11:00 PM. Our finding indicated that sleeping late on weekday significantly increased the risk of MI.

Several studies have also explored the role of late wake-up time and delayed sleep midpoint on human health. Shechter et al. found that a late wake-up time and delayed sleep midpoint could influence physical activity and decrease energy expenditure (11). A recent study also demonstrated that a late wake-up time increased the risk of congestive heart failure (26). In this study, we observed that people with a late wake-up time on weekdays had a gradually increasing incidence of MI. However, no significant associations of a late wake-up time and delaying sleep midpoint with incident MI were found in multivariate Cox regression analysis.

The specific mechanism by which sleeping late increases the risk of MI remains to be elucidated. Sleeping late may lead to impaired glucose tolerance and increased sympathetic tone, thereby increasing the prevalence of MI risk factors including diabetes mellitus, obesity and hypertension (27, 28). Delayed bedtime may also influence the circadian rhythm, and further cause endocrine disorders as well as increased fibrinogen levels and inflammation, which may promote thrombosis (29, 30). In addition, individuals with late bedtime were prone to having a shorter sleep duration, which could increase the sensitivity of the myocardium to ischemic damage and lead to early deterioration of the vascular structure and function (31, 32).

This is the first study to investigate the relationship between sleep timing and incident MI. Our results provided the evidence about the negative effect of late weekday bedtime on the incidence of MI. The present study also had some limitations. The bedtime, wake-up time, and sleep duration in the current study were self-reported based on a sleep habit questionnaire; therefore, it was difficult to avoid measurement errors and recall biases. Moreover, the current research sample was mainly composed of middle-aged and elderly white individuals, and may not be generalizable to all ages and ethnic groups.

## Conclusions

The current study established a significant association between the weekday bedtime and incident MI. Participants with weekday bedtime later than 12:00 AM had an increased incidence of MI. A suitable bedtime on weekdays may be of great significance for implementing sleep-targeted interventions to reduce the MI risk.

## Data Availability Statement

The datasets presented in this study can be found in online repositories. The names of the repository/repositories and accession number(s) can be found at: https://sleepdata.org/datasets/shhs.

## Ethics Statement

The studies involving human participants were reviewed and approved by Boston University, Case Western Reserve University, Johns Hopkins University, Missouri Breaks Research, Inc., New York University Medical Center, University of Arizona, University of California at Davis, University of Minnesota, Clinical and Translational Science Institute, University of Washington. The patients/participants provided their written informed consent to participate in this study.

## Author Contributions

BY and XM raised the idea for the study and handled supervision in our study. YF, YW, YP, BZ, JY, and LB, contributed to the study design, writing and review of the report. BY acquired the data in SHHS and participated in further data analysis. All authors approved the final version of the report.

## Funding

This study was supported by the Natural Science Basic Research Program of Shaanxi (No. 2021JQ-395) the Clinical Research Award of the First Affiliated Hospital of Xi'an Jiaotong University, China (No. XJTU1AF-CRF-2019-022).

## Conflict of Interest

The authors declare that the research was conducted in the absence of any commercial or financial relationships that could be construed as a potential conflict of interest.

## Publisher's Note

All claims expressed in this article are solely those of the authors and do not necessarily represent those of their affiliated organizations, or those of the publisher, the editors and the reviewers. Any product that may be evaluated in this article, or claim that may be made by its manufacturer, is not guaranteed or endorsed by the publisher.

## Supplementary Material

Supplement Figure 1 Multivariable adjusted restricted spline curves for the association between sleep timing and the incidence MI. **(A)**: Weekday bedtime and MI; **(B)**: Weekday wake-up time and MI.

Supplement Table 1 Hazard ratios and 95% CIs for sleep timing associated with MI on weekend.

Supplement Table 2 The interaction analysis between sleep timing and sleep duration in the final multivariable cox regression model.

## Abbreviations

AHI: apnea-hypopnea index
BMI: body mass index
MI: myocardial infarction.
