# Supplementary figures and images for: Sleeping Late Increases the Risk of Myocardial Infarction in the Middle-Aged and Older Populations
Source: Front Cardiovasc Med. 2021 Sep 24;8:709468. doi: 10.3389/fcvm.2021.709468 (PMC8498336; doi:10.3389/fcvm.2021.709468)

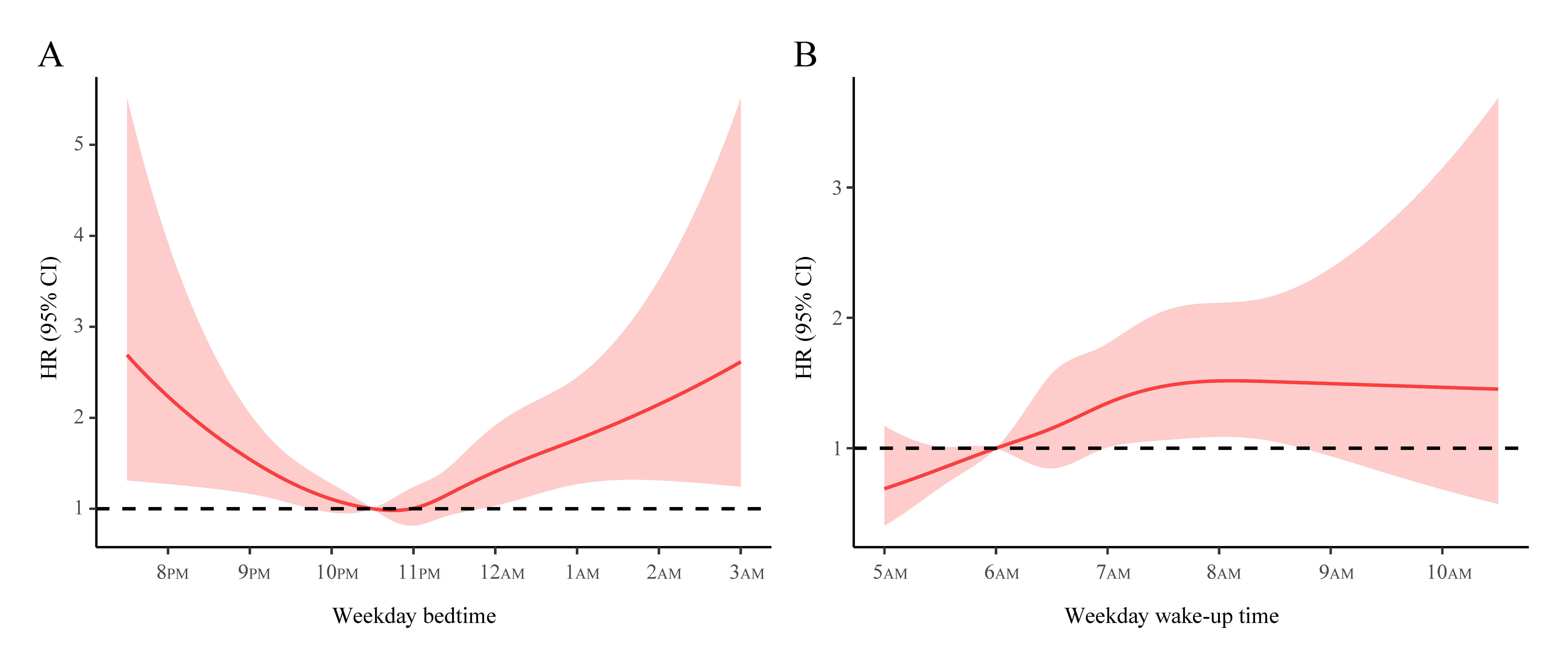

Supplement: Supplement Figure 1 — Multivariable adjusted restricted spline curves for the association between sleep timing and the incidence MI. (A): Weekday bedtime and MI; (B): Weekday wake-up time and MI. [file Image_1.TIFF]
