# Supplementary material for: Sleeping Late Increases the Risk of Myocardial Infarction in the Middle-Aged and Older Populations
Source: Front Cardiovasc Med. 2021 Sep 24;8:709468. doi: 10.3389/fcvm.2021.709468 (PMC8498336; doi:10.3389/fcvm.2021.709468)
Supplement: Supplement Table 1 — Hazard ratios and 95% CIs for sleep timing associated with MI on weekend. [file Table_1.docx]

Supplement Table 1 Hazard ratios and 95% CIs for sleep timing associated with MI on weekend

|  | **Univariate Models** |  | **Multivariable adjusted^a^** |  | **Multivariable adjusted^b^** |  |
| --- | --- | --- | --- | --- | --- | --- |
| **Sleep timing** | HR (95%CI) | P | HR (95%CI) | P | HR (95%CI) | P |
| Bedtime |  |  |  |  |  |  |
| >12:00_AM_ | 1.709 (1.199-2.435) | 0.003 | 1.514 (1.053-2.176) | 0.025 | 1.428 (0.980-2.082) | 0.064 |
| 11:01_PM_ to 12:00_AM_ | 1.194 (0.890-1.602) | 0.237 | 1.062 (0.788-1.432) | 0.693 | 1.044 (0.772-1.411) | 0.781 |
| 10:01_PM_ to 11:00_PM_ | 1 |  | 1 |  | 1 |  |
| ≤10:00_PM_ | 1.650 (1.202-2.264) | 0.002 | 1.338 (0.964-1.857) | 0.082 | 1.363 (0.977-1.901) | 0.068 |
| Wake-up time |  |  |  |  |  |  |
| >8:00_AM_ | 0.972 (0.667-1.417) | 0.884 | 1.147 (0.781-1.686) | 0.484 | 1.338 (0.864-2.072) | 0.193 |
| 7:01_AM_ to 8:00_AM_ | 0.810 (0.592-1.108) | 0.187 | 1.048 (0.760-1.444) | 0.774 | 1.178 (0.827-1.677) | 0.364 |
| 6:01_AM_ to 7:00_AM_ | 0.800 (0.602-1.062) | 0.122 | 0.881 (0.659-1.177) | 0.391 | 0.944 (0.698-1.277) | 0.710 |
| ≤6:00_AM_ | 1 |  | 1 |  | 1 |  |
| Sleep midpoint | 0.887 (0.790-0.997) | 0.043 | 0.945 (0.842-1.060) | 0.332 | 0.952 (0.848-1.068) | 0.404 |

MI, myocardial infarction; 95% CI, 95% confidence interval; HR, hazard ratio.

a adjusted for age, sex, race, BMI, smoking status, alcohol use, hypertension, diabetes mellitus, AHI

b adjusted by a+ self-reported sleep duration
