# Supplementary material for: Sleeping Late Increases the Risk of Myocardial Infarction in the Middle-Aged and Older Populations
Source: Front Cardiovasc Med. 2021 Sep 24;8:709468. doi: 10.3389/fcvm.2021.709468 (PMC8498336; doi:10.3389/fcvm.2021.709468)
Supplement: Supplement Table 2 — The interaction analysis between sleep timing and sleep duration in the final multivariable cox regression model. [file Table_2.docx]

Supplement Table 2 The interaction analysis between sleep timing and sleep duration in the final multivariable Cox regression model

| **Sleep timing and MI^&^** | **Interaction term** | **P_interaction_** |
| --- | --- | --- |
| Weekday bedtime and MI | Weekday bedtime * Sleep duration | 0.105 |
| Weekday wake-up time and MI | Weekday wake-up time * Sleep duration | 0.410 |
| Weekday sleep midpoint and MI | Weekday sleep midpoint * Sleep duration | 0.107 |
| Weekend bedtime and MI | Weekend bedtime *Sleep duration | 0.134 |
| Weekend wake-up time and MI | Weekend wake-up time * Sleep duration | 0.096 |
| Weekend sleep midpoint and MI | Weekend sleep midpoint * Sleep duration | 0.129 |

MI, myocardial infarction

**&** Multivariable Cox regression analysis adjusted for age, sex, race, BMI, smoking status, alcohol use, hypertension, diabetes mellitus, AHI and self-reported sleep duration

All the interaction term was added in the Multivariable Cox regression model, separately
